# Sex Differences in Spinocerebellar Ataxia Type 1: Clinical Presentation and Progression

**DOI:** 10.1007/s12311-025-01881-4

**Published:** 2025-07-10

**Authors:** Fabiana Colucci, Sara Stefanelli, Elena Contaldi, Andrea Gozzi, Maura Pugliatti, Pietro Antenucci, Jay Guido Capone, Daniela Gragnaniello, Mariachiara Sensi

**Affiliations:** 1 Department of Neuroscience and Rehabilitation, University of Ferrara, Ferrara, Italy; 2 Department of Clinical Neurosciences, Parkinson and Movement Disorders Unit, Fondazione IRCCS Istituto Neurologico Carlo Besta, Milan, Italy; 3 Department of Neuroscience, Azienda Ospedaliero-Universitaria S. Anna, Ferrara, Italy; 4 Centro Parkinson e Parkinsonismi ASST Gaetano Pini-CTO, Milan, Italy

**Keywords:** Spinocerebellar ataxia, SCA1, Sex, Cognition, Cognitive decline, aERPs

## Abstract

**Background:**

Spinocerebellar ataxia type 1 (SCA1) is characterised by motor and cognitive symptoms. Sex-specific differences in disease presentation and progression remain poorly understood. This study investigates the role of sex in clinical-demographic and motor/cognitive outcomes in SCA1.

**Methods:**

This single-centre, longitudinal observational cohort study was conducted at the University Hospital of Ferrara between 2021 and 2024. Consecutively, genetically confirmed SCA1 patients were evaluated at baseline and after 24±6 months. Assessments included comprehensive neuropsychological testing and auditory event-related potentials (aERPs). Motor function was evaluated using the Scale for Assessment and Rating of Ataxia (SARA).

**Results:**

Sixteen SCA1 patients (9 males, seven females) were evaluated at baseline, with 10 patients (5 males, five females) completing follow-up. Even if most cognitive functions were preserved in both sexes at baseline, males showed worse performance in emotion attribution tasks than females (42.8 ± 8.5 vs. 53.1 ± 5.7, *r* = 0.63). Over time, both sexes showed slightly worsening cognitive performance, although not statistically significant, with males demonstrating deficits in verbal fluency (*p* = 0.036) and emotion attribution (*p* = 0.048). In the same group, motor impairment worsened at follow-up, though not significantly. aERPs revealed no differences between sexes at follow-up.

**Conclusion:**

Sex may influence cognitive outcomes in SCA1, with male patients showing greater vulnerability to cognitive decline. aERPs did not show significant modifications. These findings highlight the importance of considering sex-specific approaches in the clinical management of SCA1 patients and the higher values of a comprehensive neuropsychological assessment compared to the neurophysiological approach with aERPs to reach these slight changes over time.

**Clinical trial number:**

Not applicable.

**Supplementary Information:**

The online version contains supplementary material available at 10.1007/s12311-025-01881-4.

## Introduction

Patients with cerebellar ataxia experience not only motor but also non-motor symptoms. There is growing evidence that cognitive impairment occurs during disease progression in Spinocerebellar Ataxia (SCA) patients [1–5]. Several groups have conducted specific analyses of cerebellar disease’s demographic and cognitive characteristics, with clear results for a direct association of cognitive impairment with disease duration [6]. Prediction of cognitive progression in SCA according to sex difference is more uncertain.

The sex-related cognitive decline for SCA type 1, one of the more common SCAs in Italy [7], is poorly defined and has not yet been specifically addressed in any available clinical study, notwithstanding the emerging importance of this biological variable in clinical and research studies [8, 9]. Indeed, scientific and clinical research has historically focused on male subjects, leading to gaps in understanding how neurodegenerative diseases and treatments affect females. Sex-based differences are essential for producing representative findings and developing safe, effective therapies for both sexes, particularly in the study of SCA1. Globally, limited documentation considering the sex-related differences in SCA epidemiology, phenomenology, progression, therapy responses and complications is available [10–13].

We analysed patients with SCA1 to account for the possible role of sex as a major variable in clinic-demographic features and motor and cognitive presentation, and progression.

Second, we used auditory event-related potentials (aERPs) to study cognitive processes. Studies on endogenous event-related potentials (ERPs) in spinocerebellar ataxias (SCAs) are restricted [4]. aERPs provide information about attention and memory: in the “oddball” paradigm, subjects respond only to specific target stimuli randomly presented among non-target stimuli. ERPs reflect cognitive processing: early components (N100, N200) represent sensory and perceptual processes, reflecting the automatic attention abilities in identification and response to stimuli, while the later component, P300, requires conscious attention. P300 is considered an index of active cognitive processing involving various brain areas, reflecting attention, discrimination and working memory. Indeed, P300 amplitude is directly related to the performance in memory, attention, and executive functions, while the latency to the neuronal speed to generate a response.

This approach has been applied to study cognitive changes, not only in ageing and neurodegenerative disorders [14–18] but also in cerebellar ataxias, including SCA1 and SCA2 [4, 19, 20]. However, no data are available on sex-related differences.

## Materials and methods

This single-center, longitudinal observational cohort study was conducted at the University Hospital of Ferrara between August 2021 and July 2024.

### Participants

Between July 2021 and April 2022, patients genetically diagnosed with spinocerebellar ataxia type 1 (SCA1) and referred to the Movement Disorders Centre at Ferrara Hospital were consecutively evaluated for eligibility criteria. Exclusion criteria were (i) a score > 24 on the Motor Scale for Assessment and Rating of Ataxia (SARA), and/or (ii) a lower score on Mini-Mental State Examination score (MMSE < 24), and/or hearing loss (evaluated through an audiometry exam and conducted before writing the informed content). The motor domain, assessed with SARA, and the global cognitive function, assessed with MMSE, help control for the influence of severe motor impairment and baseline cognitive deficits on the neurophysiological examination, while hearing loss may interfere with the neurophysiological test employed.

All enrolled participants were native Italian speakers and capable of providing informed consent. Participants were evaluated longitudinally: at baseline (T0) and after 24±6 months (T1).

The study protocol received approval from the local institutional review board (CE 453/2021), and all participants provided informed written consent. The study adhered to the ethical principles outlined in the 1964 Declaration of Helsinki and its subsequent amendments.

### Data Collection

We gathered information at baseline on the age of first motor cerebellar symptoms onset, the disease duration at the time of assessment, years of education, the number of CAG repeat expansions, and whether the inheritance was paternal or maternal. Additionally, at T0 and T1, information on the severity of motor symptoms was evaluated using the SARA scale [21]. The data at baseline have already been published in previous work by our group [4].

### Neuropsychological Testing

Neuropsychological assessments were administered at T0 and T1 by the same neuropsychologist (SS). The raw scores of each test were adjusted to Italian normative data for age and education (corrected score) for the analysis. To define abnormal results, the available cut-off scores were used. The assessment included:

- Mini-mental State Examination (MMSE) to briefly screen the cognitive status [22].
- Frontal Assessment Battery (FAB) to assess executive functions: conceptualisation, mental flexibility, motor programming, sensitivity to interference, inhibitory control, and environmental autonomy [23];
- Verbal fluency test (F-A-S letters) to evaluate lexical retrieval. It requires processing speed [24];
- Trail Making Test (TMT) A-B, to measure executive functions: the TMT-A assesses selective attention and motor speed, while in TMT-B, the attentional shifting [25];
- Raven Colored Progressive Matrices (RCPM) to assess non-verbal reasoning ability and visuospatial processing skills [26];
- Stroop Test, evaluating many executive functions: selective attention, sensitivity to interference and inhibitory control [27];
- Rey-Osterrieth Complex Figure (ROCF) to investigate by copying the ability of construction practice and visuospatial planning, and by recalling the visual memory [28];
- Prose memory test (Babcock’s short tale-BST) to measure the verbal-episodic memory [29];
- Emotion Attribution Task (EAT) to explore part of social cognition: emotion attribution. By 58 short stories, happiness, sadness, anger, fear, envy, embarrassment or disgust could be elicited [30];

### Electrophysiological Assessment

Auditory event-related potential (a-ERP) was performed at T0 and T1. The software used for delivering a-ERPs was KeypointTM (Natus Neurology Incorporated, Middleton, WI, USA). The assessment was conducted according to the oddball paradigm: participants wearing earphones, pseudo-randomly received at least 100 auditory stimuli (inter-stimulus interval 1200 ms), “standard” or “target”. Stimuli had the same mean sound level of 74.97 ± 3.15 dB and duration of 200 ms, but different sound frequencies, being 2000-Hz for standard stimuli and 1500-Hz for target ones. In addition, standard stimuli had a presentation probability of 80%, while target stimuli of 20% [31].

Participants should be focused on the target stimuli and this response was recorded from scalp electrodes, placed on Cz (international 10/20 system). In the assembly of the electroencephalogram, electrodes were placed at Fz, Cz and Pz, while the electrode reference was on the earlobe. We obtained values on the Peak latency and amplitude of the N100, N200, and P300 components [32].

### Statistical Analysis

Counts or percentages are used for categorical variables, and mean ± standard deviation (SD) or median and interquartile range (IQR) for continuous variables. The chi-square test or Fisher’s exact test was used to compare binary variables, while the T-test or the Mann-Whitney was applied for continuous variables according to their distribution. Holm-Bonferroni correction was applied to control for Type I errors. Effect sizes were calculated following Cohen’s guidelines, using Cohen’s “*d*” for comparisons based on t-tests and the “*r*” statistic for those based on Mann-Whitney tests. A “*d*” value of approximately 0.2 was interpreted as a small effect, around 0.5 as a medium effect, and 0.8 or above as a large effect. For “*r*”, values near 0.1, 0.3, and 0.5 were considered to indicate small, medium, and large effects, respectively.

Differences between groups were explored by analysis of covariance (ANCOVA). Multiple linear regression analysis was used to simultaneously examine how multiple independent variables (Sex, Age, Age at onset, Disease duration, CAG repeats) influence the dependent variable (motor and cognitive tests) to isolate the unique contribution of each predictor (mainly sex) while holding others constant. To track disease progression and identify factors affecting the rate of change linear mixed model was applied.

SPSS software support (IBM, v20) was used for all the statistical analyses, considering statistical significance if the results of *p* were < 0.05.

## Results

The study involved 16 SCA1 participants at baseline (T0), consisting of 9 men and 7 women. The mean (SD) age at clinical onset, the time of enrollment, years of education and thenumber of triplets carrying the ataxin-1 gene was similar between sexes without any statistically significant differences. Overall, participants have mild-to-moderate motor deficits, assessed by the SARA scale: 12.2 (4.2) in males and 10.7 (6.4) in female patients (*p* = 0.46) Table 1.

**Table 1:** Clinical-demographic characteristics of participants at the baseline (T0) and at Follow-up (T1)

	T0			T1		
	Males	Females	*p*	Males	Females	*p*
N (%)	9 (56.3)	7 (43.8)	*-*	5 (50.0)	5 (50.0)	*-*
Onset age, mean (SD)	41.8 (7.0)	40.7 (9.8)	*0.81*	39.6 (4.6)	43.4 (7.3)	*0.48*
Age at enroll, mean (SD)	47.8 (6.9)	48.1 (10.2)	*0.94*	*48.7 (6.1)*	*53.3 (6.8)*	*0.36*
Education, mean (SD)	10.6 (2.5)	12.0 (3.5)	*0.37*	11.0 (2.7)	10.6 (2.5)	*0.81*
Disease Duration, mean (SD)	6.0 (3.9)	7.4 (4.0)	*0.48*	10.2 (3.8)	10.8 (3.9)	*0.93*
CAG expansion, mean (SD)	49.8 (7.9)	45.3 (3.4)	*0.23*	51.8 (9.1)	45.2 (3.3)	*0.25*
SARA scale	12.2 (4.2)	10.7 (6.4)	*0.46*	18.8 (6.8)	14.0 (6.5)	*0.30*

**Fig. 1:**
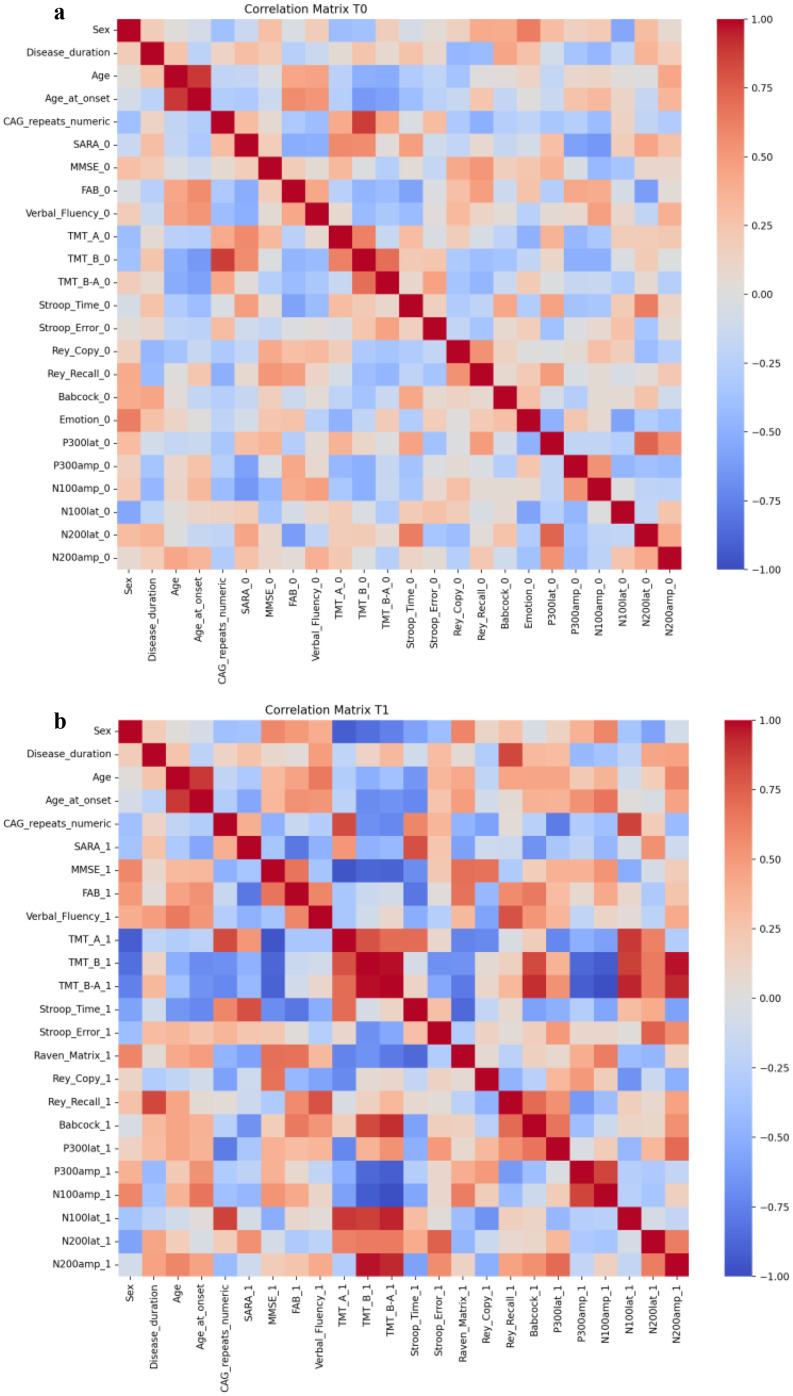
Correlation matrix at T0 **(a)** and T1 **(b)** evaluated the influence of sex, disease duration, age, age at onset and CAG repeats on the motor, cognitive scores and aERP

Table 2 reports data on neuropsychological assessment at T0 according to sex. Overall, both males and females showed scores in normal ranges, except in emotion attribution tasks, where male patients showed pathological cut-off and a worse performance compared to females, although not significant after correction for multiple testing[mean (SD) EAT: 42.8 (8.5) males vs. 53.1 (5.7) females, p-value adjusted for age 0.04 [effect size = 0.38 (medium); *p* = 0.87] after Holm-Bonferroni correction]. Multiple regression analysis, using sex, age, age at onset, disease duration, and CAG repeats as independent variables to evaluate motor and cognitive profile, showed that sex had a strong correlation with EAT [*r* = 0.627 (large effect size); *p* = 0.029] (Fig. 1a, Supplemental Table 1).

**Table 2:** Mean (SD) obtained at each neuropsychological assessment test at baseline (T0), reported according to sex. *p*^*a*^*T-test; p*^*b*^: adjusted for age, p^c^:*Holm-Bonferroni correction*

Test (pathological cut-off)	Males *N* = 9	Females *N* = 7	*p* ^a^	*p* ^b^	*p* ^c^
MMSE (< 24)	29.1 (1.0)	29.6 (0.5)	*0.29*	*0.32*	*1.00*
FAB (< 13.4)	15.9 (1.3)	15.8 (1.8)	*0.93*	*0.90*	*1.00*
Verbal fluency (< 17.35)	24.4 (9.7)	24.6 (1.0)	*0.48*	*0.47*	*1.00*
RCPM (< 18.96)	31.0 (2.6)	30.4 (4.1)	*0.74*	*0.64*	*1.00*
Test Stroop (errors) (> 4.24)	0.36 (0.9)	0.42 (0.9)	*0.88*	*0.85*	*1.00*
Test Stroop (time) (> 36.92)	22.9 (10.0)	21.9 (8.0)	*0.84*	*0.89*	*1.00*
TMT A (> 94)	80.0 (24.5)	57.8 (15.1)	*0.09*	*0.16*	*1.00*
TMT B (> 187)	135.7 (36.1)	108.6 (35.4)	*0.20*	*0.25*	*1.00*
TMT B-A (> 187)	40.7 (32.9)	51.1 (31.0)	*0.55*	*0.39*	*1.00*
ROCF (< 28.53)	33.5 (3.2)	34.1 (1.3)	*0.61*	*0.40*	*1.00*
ROCF– recall (< 9.46)	11.2 (5.4)	15.5 (5.0)	*0.16*	*0.17*	*1.00*
BST (< 8.2)	7.7 (2.6)	10.3 (3.8)	*0.16*	*0.16*	*1.00*
EAT (< 44.19)	*42.8 (8.5)*	53.1 (5.7)	***0.029***	***0.04***	*0.87*

At T1, six patients dropped out: two due to difficulties in reaching the center with caregivers, and four refused to undergo further neuropsychological and neurophysiological assessments, which they considered lengthy and tiring. Data were collected from the remaining 10 patients, evenly divided between both sexes (Table 1). The male patients (*n* = 5) who continued in the follow-up had a mean (SD) age at clinical onset of 39.6 (4.6) years and disease duration of 10.2 (3.8) years. Female patients showed similar results, with a mean (SD) age at clinical onset of 43.4 (7.3) years and disease duration of 10.8 (3.9) years. The mean (SD) years of education were 11.0 (2.7) for men and 10.6 (2.5) for women.

The number of triplets repeats in the ataxin-1 gene was higher in males [mean (SD): 51.8 (9.1)] compared to females [45.2 (3.3)], though this difference was not statistically significant.

At follow-up, males continued to display moderate motor impairment, which was greater than in females, albeit without statistical significance [mean (SD) SARA scale: males 18.8 (6.8) vs. females 14.0 (6.5)]. The linear mixed model showed a significant negative effect of sex on SARA change (*r* = 0.751; *p* = 0.008).

**Table 3:** Mean (SD) obtained at each neuropsychological assessment test at T1, reported according to sex. *p*^*a*^*T-test; p*^*b*^: adjusted for age, p^c^:*Holm-Bonferroni correction*

Test (pathological cut-off)	Males *N* = 5	Females *N* = 5	*p* ^a^	*p* ^b^	*p* ^c^
MMSE (< 24)	29.0 (1.2)	29.6 (0.5)	*0.16*	*0.18*	*1.00*
FAB (< 13.4)	15.2 (1.7)	16.6 (1.5)	*0.25*	*0.33*	*1.00*
Verbal fluency (< 17.35)	16.6 (4.8)	27.3 (6.1)	***0.002***	*0.003*	***0.036***
RCPM (< 18.96)	31.5 (3.0)	31.5 (4.5)	*0.07*	*0.13*	*1.00*
Test Stroop (errors) (> 4.24)	0.75 (1.3)	0.60 (1.0)	*0.28*	*0.10*	*1.00*
Test Stroop (time) (> 36.92)	32.4 (6.8)	18.0 (5.2)	***0.01***	***0.04***	*0.48*
TMT A (> 94)	75.7 (25.7)	49.8 (6.6)	***0.03***	*0.03*	*0.36*
TMT B (> 187)	139.7 (36.1)	101.8 (39.1)	*0.16*	*0.39*	*1.00*
TMT B-A (> 187)	48.0 (43.2)	52.6 (36.8)	*0.42*	*0.49*	*1.00*
ROCF (< 28.53)	32.3 (3.3)	34.2 (1.5)	*0.87*	*0.71*	*1.00*
ROCF– recall (< 9.46)	7.0 (6.2)	13.7 (2.9)	*0.05*	*0.05*	*0.39*
BST (< 8.2)	9.7 (1.9)	10.5 (4.3)	*0.91*	*0.56*	*1.00*
EAT (< 44.19)	44.0 (4.3)	55.8 (1.5)	***0.003***	*0.004*	***0.048***

Table 3 shows neuropsychological assessment data at T1 in the 10 patients who performed follow-up.

All tests resulted in normal ranges in females, while males showed deficits in verbal fluency test [mean (SD), 16.6 (4.8)], recall of Copy Rey Figure [mean (SD), 7.0 (6.2)], and, still, in emotional attribution tasks [mean (SD), 44.0 (4.3)]. Comparing each test between sexes, females performed better on the verbal fluency test [mean (SD), males 16.6 (4.8) vs. females 27.3 (6.1), *p* = 0.036 after Holm-Bonferroni correction, effect size 0.15], and emotion attribution tasks [mean (SD) males 44.0 (4.3) vs. females 55.8 (1.5), *p* = 0.048 after Holm-Bonferroni correction, effect size = 0.32 (medium)].

TMT-A [mean (SD) males 75.7 (25.7) vs. females 49.8 (6.6), effect size = 0.83 (large)], Stroop test in terms of time [mean (SD) males 32.4 (6.8) vs. females 18.0 (5.2), effect size = 0.39 (medium)] and recall of Copy Rey Figure [mean (SD) males 7.0 (6.2) vs. females 13.7 (2.9) effect size = 0.25 (small)] were better performed in females although without any significant after Holm-Bonferroni corrections for multiple testing.

Multiple regression analysis, using sex, age, age at onset, disease duration, and CAG repeats as independent variables to evaluate motor and cognitive profile at T1, showed that sex influences TMT-A (*r*=-0.92; *p* = 0.027) (Fig. 1b, Supplemental Table 1). SARA score showed a strong correlation with EAT (*r*=-0.919), FAB (*r*=-0.79), BST (*r*=-0.668).

Supplemental Table 2 presents the raw scores—uncorrected for age and education—for each test, along with the percentage of patients showing pathological results at specific time points (T0: baseline; T1: follow-up). Supplemental Table 3 provides an example of raw and corrected score calculations at baseline for one of the enrolled patients. Table 4 displays the differences in corrected scores between T1 and T0 from the neuropsychological assessment, stratified by sex. A slight, non-significant decline in performance is observed across all tests in both sexes.

**Table 4:** Mean difference (SD) of scores obtained at each neuropsychological assessment test at T1 vs. T0, reported according to sex. *p*^*a*^: *T-test; p*^*b*^: adjusted for age (ANCOVA)

Test	Male	Famale	*p* ^a^	*p* ^b^
MMSE	-1.3 (2.3)	-0.2 (0.8)	*0.50*	*0.50*
FAB	-0.6 (0.9)	0.2 (1.5)	*0.36*	*0.57*
Verbal fluency	-3.6 (2.6)	5.0 (7.5)	*0.66*	*0.82*
RCPM	-2.5 (5.3)	2.2 (5.0)	*0.23*	*0.23*
Test Stroop (errors)	0.8 (2.1)	0.6 (1.0)	*0.78*	*0.48*
Test Stroop (time)	2.7 (13.4)	-2.4 (8.1)	*0.99*	*0.62*
TMT A	7 (6.2)	9 (8.1)	*0.89*	*0.76*
TMT B	17 (12.6)	18 (13)	*0.92*	*0.67*
TMT B-A	16 (13.7)	18.6 (15.3)	*0.82*	*0.86*
ROCF	0.2 (1.2)	-0.3 (2.0)	*0.47*	*0.68s*
ROCF– recall	-1.9 (4.7)	1.2 (5.8)	*0.65*	*0.73*
BST	-1.1 (5.1)	-2.1 (2.9)	*0.72*	*0.25*
EAT (< 44.19)	-2.1 (3.4)	-2.2 (3.1)	*0.97*	*0.89*

**Fig. 2:**
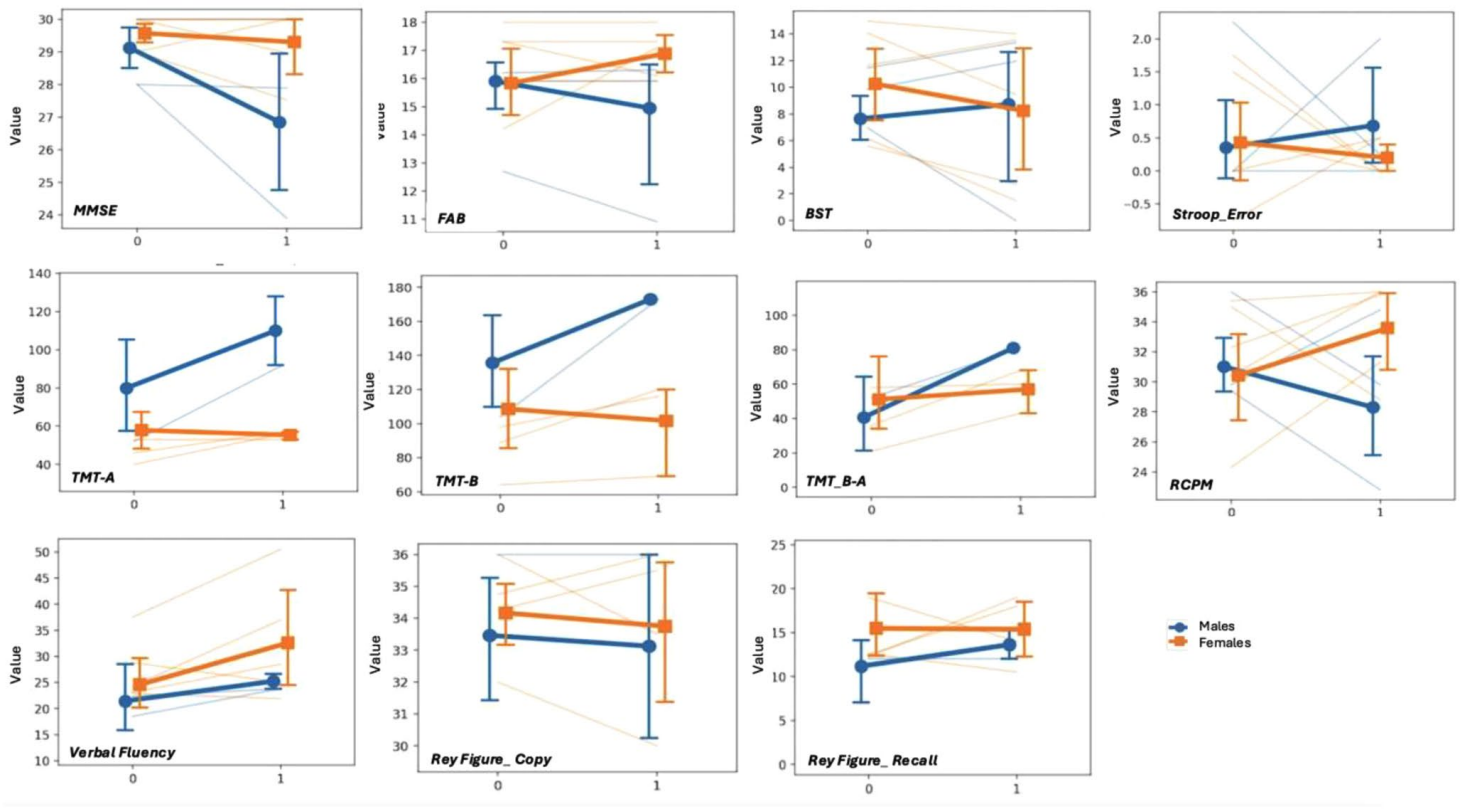
Linear mixed model according to sex for each cognitive test. 0 indicate the baseline values, 1 the follow-up

Mixed linear model showed only a marginally statistically significant influence of sex on TMT B-A (*r* = 0.876; *p* = 0.051) and MMSE changes (*r* = 0.766; *p* = 0.064) (females showed slower decline). However, a strong effect of sex was also detected on TMT B (*r* = 0.789), TMT A (*r* = 0.753) and verbal fluency (*r* = 0.621). Figure 2. In addition, the model showed that disease duration had a strong positive correlation with Rey Figure Recall changes (*r* = 0.966) while CAG repeats with Stroop Time changes (*r* = 0.911) (Supplemental Fig. 1).

**Fig. 3:**
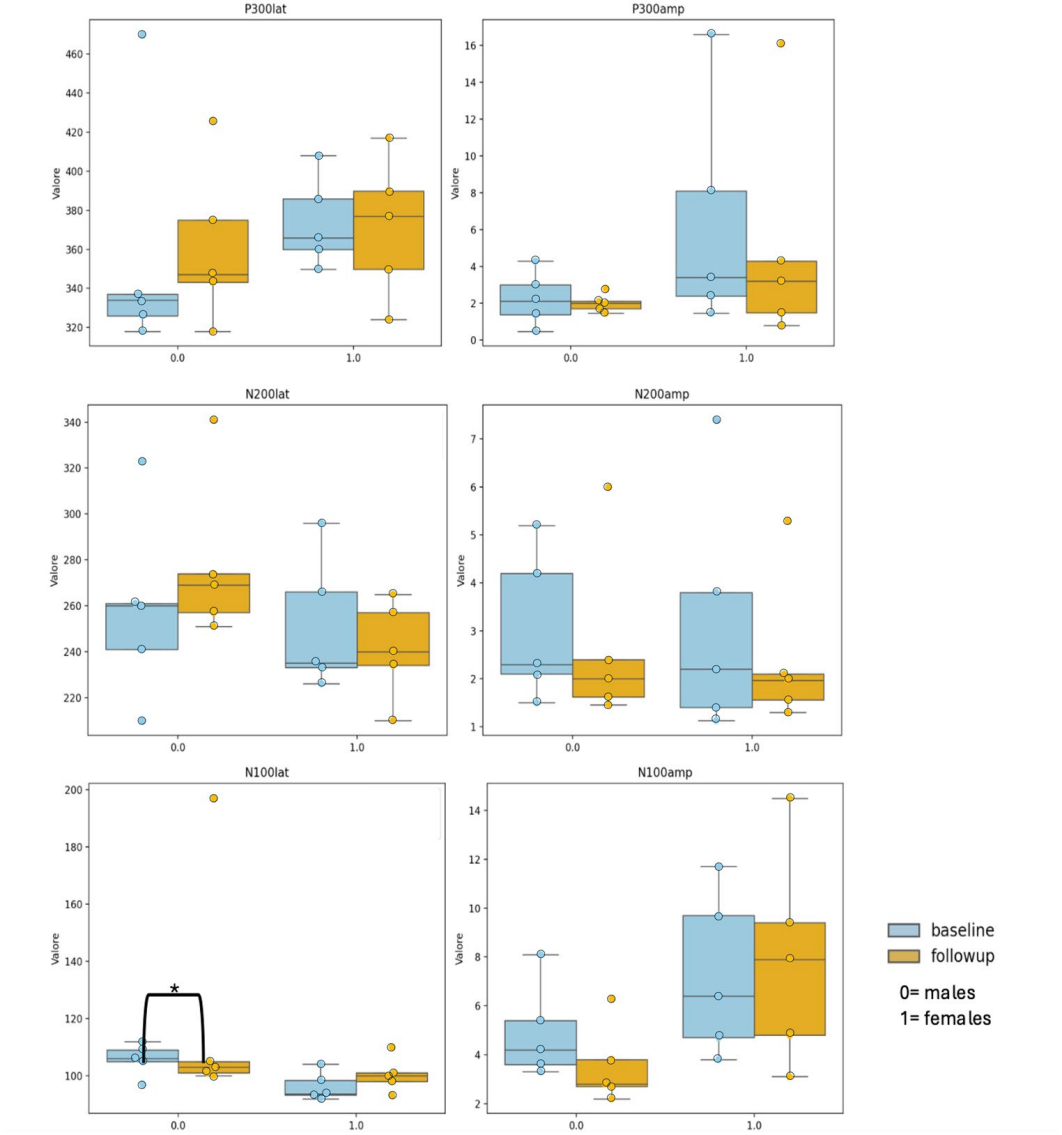
Event-related potentials: principal components at T0 (0) and T1 (1) [of which latency (lat) and amplitude (amp) were measured] by sex. * Indicated a p-value < 0.05, according to t-test and adjusted for age (ANCOVA)

Analysis of ERPS, Fig. 3 shows the latencies and amplitudes data of aERPs in the 10 patients who underwent both neuropsychological examinations (T0 and T1). No differences were detected in the two timelines in each sex group. The differences between sex at baseline and follow-up of aERP revealed only a higher N100 latency in females compared to males at baseline [mean (SD) males vs. females, *p* = 0.034, effect size = 0.31 (small)]; no further differences were detected at baseline and follow-up. Multiple regression analysis showed that sex has a negative correlation with N100 latency (r-0.55; *p* = 0.0280) (Fig. 1, Supplemental Table 1).

The mixed linear model showed no significant influence of sex on wave changes. However, it showed that disease duration had a negative correlation with N200amp change (*r*=-0.653), CAG repeats had a strong positive correlation with N100lat change (*r* = 0.845) and Age at onset and Age both positively correlated with N100amp changes (*r* ≈ 0.65–0.67) (Supplemental Fig. 1).

## Discussion

### Clinical progression

Of the 16 patients (nine male) initially enrolled in the study at baseline, 10 (five male) were included in the follow-up assessment at 24±6 months. In terms of age of onset (approximately 41 years), SCA1 affects both males and females similarly, consistent with previous studies [11, 12]. Interestingly, although not statistically significant, a tendency for earlier disease onset in males compared to females was noted, with males typically presenting symptoms towards the end of the fourth decade of life, while females tend to experience onset at the beginning of the fifth decade. This is in line with Du Montcel and colleagues, who demonstrated a 5-year earlier presentation of ataxia symptoms in males compared to females [11]. However, the literature does not consistently report clear sex-based differences in the age of onset or disease severity, though it is well-established that these factors are correlated with the size of the expanded alleles [12]. Each additional repeat caused the largest reduction in age at the onset of 0.049 years [12]. Indeed, men in the present cohort exhibited a higher average number of CAG triplet expansions compared to women (51.8 vs. 45.2), and this might explain the earlier age at presentation of symptoms. However, the gender effects on the age of onset and mean CAG repeats were not so clear and not confirmed in other studies [33]. Indeed, Riess et al., in a cohort of SCA1 and SCA3 patients, noted gender effects in SCA patients on transmission rather than clinical expression [33].

Regarding motor impairment and disease progression, in the present study, both sexes presented with mild-to-moderate motor deficits at baseline as measured by the SARA scale. According to SCA type, sex differences in disease motor progression should be carefully considered. In SCA6, previous research by Jacobi et al. found that the female sex is associated with faster progression, with a hazard ratio of 1.7 (95% CI: 1.1–2.6) for progression to more advanced disability stages [12]. Conversely, our data suggests a potential opposite trend in SCA1, with males potentially experiencing faster motor deterioration, although this difference didn’t reach statistical significance in our small cohort. However, our findings confirm results on preclinical models [34]. Indeed, Selimovic et al. detected a significantly higher GFAP intensity in female mice, indicating more severe astrocyte reactivity, contributing to excitotoxicity, altered neurotransmitter uptake, and disruption of the blood-brain barrier. Conversely, male mice exhibited significantly higher microglial density [34]. This difference in glial cell activation patterns likely contributes to the sex-specific disease progression through different neuroinflammatory mechanisms [34].

This contrasting pattern suggests that the influence of sex on disease progression may vary according to SCA subtype, potentially reflecting differences in underlying pathophysiological mechanisms or sex-specific modifiers of disease expression. Beyond the specific differences among the various SCA subtypes, our findings confirm the well-documented clinical progression of cerebellar motor dysfunction in patients with SCA, representing a fundamental characteristic of the pathology, regardless of potential variables that might modulate its rate or expression [12, 35].

The clinical progression in patients with SCA was globally analysed by Weber and colleagues, who combined data from two major European cohorts with a three-year follow-up focused on health-related quality of life progression [13]. The research revealed that quality of life decline was particularly pronounced in males with younger disease onset (before age 40) and with a BMI between 30 and 35 [13]. Generally, with disease progression, quality of life is influenced by increasingly severe cerebellar problems (i.e. dysarthria, dysphagia) and mild cognitive problems [10]. However, the progression of cognition according to sex has been less analysed. Indeed, the present study focused mainly on this aspect, showing that affected patients, even 10 years after disease onset, presented neuropsychological test scores within normal limits at baseline. However, a deterioration in scores, albeit within normal limits, was observed across all tests during follow-up, indicating changes in cognitive performance. These findings support the notion that progressive cerebellar and neuronal degeneration in SCA1 affects not only motor functions but also cognitive domains [1, 4]^36^– [40]. When considering cognitive and emotional processing differences between males and females with SCA1, this study showed that males performed worse than females in emotion attribution tasks, with statistically significant differences at follow-up, suggesting a specific deficit in social cognition affecting male SCA1 patients more severely. However, the strongest correlation with emotional processing was observed with SARA scores, rather than sex. The higher SARA scores in males may account for this association. Nonetheless, in other neurological conditions involving cerebellar pathways during neurodevelopment, such as autism, males often exhibit reduced emotional discrimination compared to females [41], suggesting a potential sex-specific neurobiological difference.

In addition, at follow-up, females outperformed men on the verbal fluency test with a mediaum/large effect (*r* = 0.621). It is important to note that the verbal fluency test assesses not only language and executive functions but also cognitive flexibility, specifically evaluating the patient’s ability to access their lexical knowledge [24]. Literature indicates that patients with higher SARA scores often show an inverse correlation with verbal fluency test scores [4]. This suggests that motor involvement, particularly dysarthria, may influence verbal fluency as measured by this test. In our cohort, men demonstrated a more severe clinical presentation, which could partly explain this result; however, SARA negative correlated with FAB and BST, but not verbal fluency in our small sample. It is worth noting that, unfortunately, there was a reduction in the number of participants at follow-up, which reduced the statistical power of the analysis.

Evaluating the changes during follow-up, the main influence with a large effect of sex on progression was on TMT A (*r* = 0.753), TMT B (*r* = 0.789), TMT B-A (*r* = 0.876; *p* = 0.051) and MMSE (*r* = 0.766; *p* = 0.064), describing a female’s slower decline.

These findings support the notion that cerebellar disorders like SCA1 affect cognitive domains beyond motor control [42], with a particular impact on executive functions and emotional processing, and our data suggests these networks may be differently affected in males versus females with SCA1. The literature reported that volumetric MRI exhibited a smaller cerebellum volume and faster age-dependent volume reduction in males than females in both health and neurodegenerative disorders (i.e. mild cognitive impairment, Alzheimer’s Disease, Parkinson’s Disease) [43], which might explain the different sex progression of cognitive tests. In addition, neuroinflammation differs between sexes, and estrogens have a neuroprotective effect, mainly on spatial memory [44]. However, in a preclinical mouse model, it seems that cognitive deficits progress differently between sexes during the disease, with an early pronounced cognitive impairment in males and more severe deficits, particularly in spatial memory, in advanced female mice [33]. However, humans and mice differ in estrogen levels, and mice do not present menopause [33]; therefore, further studies are necessary to understand the role of hormones in SCA1 patients, being in the present study includes females in menopause.

Finally, genetic factors contribute substantially to gender disparities. Genes located on the sexual chromosomes influence mitochondrial metabolism and neuronal resilience, affecting how neurons respond to pathological stress [45].

### Neurophysiological progression

In our analysis, event-related potentials were recorded in response to auditory stimuli (aERPs). After processing, we examined various ERP components (P300, N100, N200) in terms of latency and amplitude. No significant differences were observed in the aERPs between T0 and T1 for the entire sample. However, when comparing the sexes, N100 latency was higher in males at baseline. This component is associated with early sensory processing and attention allocation. The lack of significant neurophysiological differences at follow-up, despite clear cognitive differences, suggests that functional compensation mechanisms might be at play, particularly in female patients.

To our knowledge, no studies in the literature have investigated neurophysiological follow-up via ERPs in a homogeneous population of SCA1 patients and the correlation with sex. Longitudinal studies often involve heterogeneous populations across various SCA genotypes [12].

The main limitations of the study are: (i) the small sample size, particularly at follow-up (*n* = 10), limits statistical power and generalizability, and (ii) the high dropout rate (37.5%) might introduce selection bias, as patients with more severe symptoms might have been more likely to discontinue. Our small sample size could influence our results, both in reaching false-positive and false-negative results. Future studies should aim to recruit larger cohorts with balanced sex representation and investigate potential protective factors that might explain the relative preservation of cognitive functions in female SCA1 patients. The relationship between CAG repeat length and cognitive outcomes also warrants further investigation. In addition, a longer follow-up period (> 24 months) may likely be needed to predict progression toward a dementia-like state and the application of quality-of-life scales (i.e. Activities of Daily Living/ADL, Patient Health Questionnaire-9/PHQ-9, EuroQol 5 Dimensions, 3 Levels /EQ-5D-3 L and EuroQol Visual Analogue Scale/EQ-VAS could better detect the global progression.

## Conclusion

This study provides preliminary evidence for sex-based differences in cognitive and emotional processing in SCA1 patients, with males showing greater vulnerability. While motor progression appears to affect both sexes, the pattern of cognitive decline may be influenced by sex-specific factors (i.e. brain structure, hormones, genetics, neuroinflammations) that deserve further investigation. Regarding the neurophysiological findings studied through the use of event-related auditory potentials, no sex differences were found between T0 and T1, indicating that neurophysiological examination does not represent a reliable test for assessing the evolution of cognitive impairment in this patient group in all populations, in each sex and between sexes.

The documented gender differences highlight the importance of considering gender as a significant factor in clinical assessment and the need for gender-specific therapeutic approaches, with potentially greater attention to interventions targeting motor and cognitive symptoms in males. However, it’s important to note that these are general trends, and individual patient care should always be tailored to specific needs regardless of gender.

## Electronic Supplementary Material

Below is the link to the electronic supplementary material.

Supplementary Material 1

## Data Availability

The data supporting the findings of this study are available on request from the corresponding author. The data are not publicly available due to privacy and ethical restrictions.
