# Correction to: Sex Differences in Spinocerebellar Ataxia Type 1: Clinical Presentation and Progression

**DOI:** 10.1007/s12311-025-01907-x

**Published:** 2025-09-03

**Authors:** Fabiana Colucci, Sara Stefanelli, Elena Contaldi, Andrea Gozzi, Maura Pugliatti, Pietro Antenucci, Jay Guido Capone, Daniela Gragnaniello, Mariachiara Sensi

**Affiliations:** 1 Department of Neuroscience and Rehabilitation, University of Ferrara, Ferrara, Italy; 2 Department of Clinical Neurosciences, Parkinson and Movement Disorders Unit, Fondazione IRCCS Istituto Neurologico Carlo Besta, Milan, Italy; 3 Department of Neuroscience, Azienda Ospedaliero- Universitaria S. Anna, Ferrara, Italy; 4 Centro Parkinson e Parkinsonismi ASST Gaetano Pini-CTO, Milan, Italy

**Correction to: The Cerebellum (2025) 24: article number 127**

10.1007/s12311-025-01881-4

The article “Sex Differences in Spinocerebellar Ataxia Type 1: Clinical Presentation and Progression”, written by Fabiana Colucci, Sara Stefanelli, Elena Contaldi, Andrea Gozzi, Maura Pugliatti, Pietro Antenucci, Jay Guido Capone, Daniela Gragnaniello & Mariachiara Sensi, was originally published electronically on the publisher’s internet portal on 10 July 2025 without open access. With the author(s)’ decision to opt for Open Choice the copyright of the article changed on 20 August 2025 to © The Author(s) 2025 and the article is forthwith distributed under a Creative Commons Attribution 4.0 International License, which permits use, sharing, adaptation, distribution and reproduction in any medium or format, as long as you give appropriate credit to the original author(s) and the source, provide a link to the Creative Commons licence, and indicate if changes were made. The images or other third party material in this article are included in the article’s Creative Commons licence, unless indicated otherwise in a credit line to the material. If material is not included in the article’s Creative Commons licence and your intended use is not permitted by statutory regulation or exceeds the permitted use, you will need to obtain permission directly from the copyright holder. To view a copy of this licence, visit http://creativecommons.org/licenses/by/4.0.

