# Supplementary material for: Sex Differences in Spinocerebellar Ataxia Type 1: Clinical Presentation and Progression
Source: Cerebellum. 2025 Jul 10;24(5):127. doi: 10.1007/s12311-025-01881-4 (PMC12405387; doi:10.1007/s12311-025-01881-4)
Supplement: Supplementary file 1 — Supplementary Material 1 [file 12311_2025_1881_MOESM1_ESM.docx]

**Supplemental Table 1.** Relevant correlation at T0 and T1 evaluated the influence of sex, disease duration, age, age at onset, and CAG repeats on cognitive scores and aERP.

| ***Outcome*** | ***Predictor*** | ***Correlation*** |
| --- | --- | --- |
| **T0** | | |
| TMT B | CAG repeats | 0,86951 |
| TMT B | Age at onset | -0,62771 |
| Emotion | Sex | 0,626996 |
| TMT B-A | Age at onset | -0,57948 |
| FAB | Age at onset | 0,558406 |
| N100lat | Sex | -0,54807 |
| TMT B-A | Age | -0,5199 |
| Verbal Fluency | Age at onset | 0,519071 |
| TMT B | Age | -0,50761 |
| **T1** | | |
| TMT A | Sex | -0,91936 |
| N100lat | CAG repeats | 0,852513 |
| Rey Recall | Disease duration | 0,843319 |
| TMT B | Sex | -0,83873 |
| TMT A | CAG repeats | 0,82232 |
| P300lat | CAG repeats | -0,77631 |
| TMT B-A | Sex | -0,75493 |
| TMT B-A | CAG repeats | -0,71472 |
| Stroop Time | Age at onset | -0,71215 |
| TMT B | Age at onset | -0,70292 |

**Supplemental Table 2.** Raw scores for each test and percentage of patients with pathological results at specific time points (T0: baseline, T1: follow-up).

|  | **T0** | | **T1** | |
| --- | --- | --- | --- | --- |
| **Test**  **(% with pathological results)** | **Males**  **N=9** | **Females**  **N=7** | **Males**  **N=5** | **Females**  **N=5** |
| **MMSE**  **(%<24)** | 29.1 (1.0)  - | 29.6 (0.5)  - | 27.8 (2.2)  - | 29.2 (1.3)  - |
| **FAB**  **(%<13.4)** | 16.4 (1.0)  - | 16.6 (1.3)  - | 16.0 (2.7)  20.0% | 17.0 (0.7)  - |
| **Verbal fluency**  **(%<17.35)** | 25.3 (2.3)  33.3% | 31.6 (10.6)  14.3% | 23.7 (8.6)  - | 26.7 (4.5)  - |
| **RCPM**  **(%<18.96)** | 31.3 (2.7)  - | 32.6 (2.8)  - | 29.4 (3.3)  - | 33.8 (3.3)  - |
| **Test Stroop (errors) (%>4.24)** | 0.29 (0.3)  - | 0.43 (1.0)  - | 0.19 (0.6)  - | 0.20 (0.3)  - |
| **Test Stroop (time) (%>36.92)** | 20.2 (9.7)  11.1% | 19.0 (6.7)  - | 21.5 (11.3)  - | 14.9 (4.0)  - |
| **TMT A**  **(%>94)** | 84.3 (35.4)  33.3% | 61.1 (13.0)  - | 108.5 (23.3)  20.0% | 62.7 (9.5)  - |
| **TMT B**  **(%>187)** | 145.2 (41.1)  11.1% | 118.4 (31.6)  - | 169.0 (34.2)  - | 121.7 (31.2)  - |
| **TMT B-A**  **(%>187)** | 54.3 (21.6)  - | 60.0 (32.0)  - | 77.0 (19.3)  - | 71.7 (22.3)  - |
| **ROCF**  **(%<28.53)** | 33.7 (3.0)  - | 34.5 (1.8)  - | 34.0 (2.3)  - | 33.8 (3.2)  - |
| **ROCF – recall**  **(%<9.46)** | 13.9 (6.7)  11.1% | 15.0 (6.5)  14.3% | 17.5 (2.1)  - | 12.0 (5.7)  20.0% |
| **BST**  **(%<8.2)** | 8.6 (2.9)  44.4% | 11.4 (4.0)  28.6% | 13.2 (1.9)  - | 9.2 (5.6)  40.0% |
| **EAT**  **(%<44.19)** | *42.8 (8.5)*  *33.3%* | 53.1 (5.7)  14.3% | 44.0 (4.3)  *40%* | 55.8 (1.5)  *20%* |

Data are expressed as mean (standard deviation) of the raw scores obtained from each test. The percentage of pathological results was estimated based on the number of patients who completed the test with pathological results divided by the total number of patients who completed the test. *Abbreviation:* BST: Babcock’s short tale; EAT: Emotion Attribution Task; FAB: Frontal Assessment Battery; MMSE: Mini-mental State Examination; RCPM: Raven Colored Progressive Matrices ROCF: Rey-Osterrieth Complex Figure; TMT: Trail Making Test A-B.

**Supplemental Table 3.** Uncorrected (raw-r), correction and corrected (c) scores of a study participant (Patient ID SCA01, Age 54 y.o., Sex: female, Education: 13 years) calculated according to Italian normative standards. *Abbreviation:* BST: Babcock’s short tale; FAB: Frontal Assessment Battery; MMSE: Mini-mental State Examination; RCPM: Raven Colored Progressive Matrices ROCF: Rey-Osterrieth Complex Figure; TMT: Trail Making Test A-B.

| **Test** | **Raw score** | **Correction (^reference)^** | **Corrected score** |
| --- | --- | --- | --- |
| **MMSE** | 30 | 0^1^ | 30 |
| **FAB** | 15 | -0,80^2^ | 14,20 |
| **Verbal Fluency** | 28 | -4^3^ | 24 |
| **RCPM** | 35 | -2,20^4^ | 32,80 |
| **Stroop (Time)** | 21 | 1,75^5^ | 22,75 |
| **Stroop (Errors)** | -1 | 0,25^5^ | -0,75 |
| **TMT A** | 51 | 2^6^ | 53 |
| **TMT B** | 85 | 5^6^ | 90 |
| **TMT B-A** | 34 | 2^6^ | 36 |
| **ROCF** | 35 | -0,75^7^ | 34,25 |
| **ROCF Recall** | 13 | 0,25^7^ | 13,25 |
| **BST** | 16,20 | -1,25^8^ | 14,95 |

**Supplemental Figure 1.** Linear mixed model: age, age at onset, disease duration, sex and CAG repeats were used as independent variables, while each cognitive test and SARA changes (T1-T0) were evaluated as dependent variables. CAG repeats are directly correlated with the Stroop test (time) and N100 latency changes, and disease duration is directly correlated with the Figure Rey recall and inversely with N200 amplitude changes. Age and age at onset are directly correlated with N100 amplitude changes. *Abbreviation:* FAB: Frontal Assessment Battery; MMSE: Mini-mental State Examination; SARA: Scale for Assessment and Rating of Ataxia; TMT: Trail Making Test A-B.


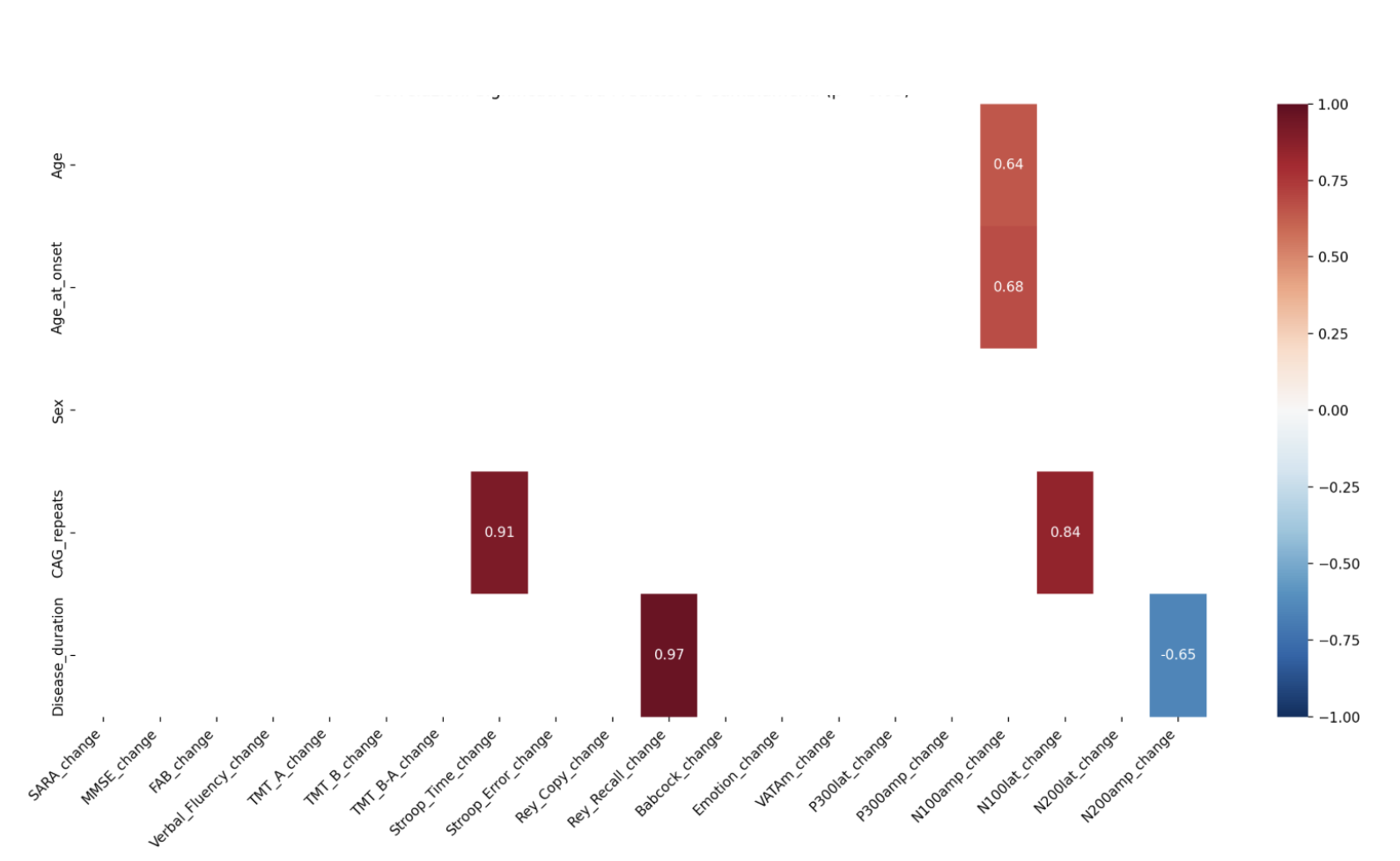


***References***

1. Foderaro G, Isella V, Mazzone A, Biglia E, Di Gangi M, Pasotti F, Sansotera F, Grobberio M, Raimondi V, Mapelli C, Ferri F, Impagnatiello V, Ferrarese C, Appollonio IM. Brand new norms for a good old test: Northern Italy normative study of MiniMental State Examination. Neurol Sci. 2022 May;43(5):3053-3063. doi: 10.1007/s10072-021-05845-4. Epub 2022 Jan 6. Erratum in: Neurol Sci. 2024 Nov;45(11):5563-5564. doi: 10.1007/s10072-024-07585-7. PMID: 34989910; PMCID: PMC9018649.

2. Appollonio I, Leone M, Isella V, Piamarta F, Consoli T, Villa ML, Forapani E, Russo A, Nichelli P. The Frontal Assessment Battery (FAB): normative values in an Italian population sample. Neurol Sci. 2005 Jun;26(2):108-16. doi: 10.1007/s10072-005-0443-4. PMID: 15995827.

3. Novelli, G., et al. "Tre test clinici di ricerca e produzione lessicale. Taratura su sogetti normali." Archivio di psicologia, neurologia e psichiatria (1986).

4. Belacchi, Carmen, et al. "Matrici progressive di Raven forma colore (CPM-47). Manuale d’uso e standardizzazione italiana." (2008).

5. Caffarra P, Vezzadini G, Dieci F, Zonato F, Venneri A. (2002). A short version of the Stroop test: normative data in an Italian population sample. Nuova Rivista di Neurologia, 12(4), 111-115.

6. Siciliano M, Chiorri C, Battini V, Sant'Elia V, Altieri M, Trojano L, Santangelo G. Regression-based normative data and equivalent scores for Trail Making Test (TMT): an updated Italian normative study. Neurol Sci. 2019 Mar;40(3):469-477. doi: 10.1007/s10072-018-3673-y. Epub 2018 Dec 7. PMID: 30535956.

7. Caffarra P, Vezzadini G, Dieci F, Zonato F, Venneri A. Rey-Osterrieth complex figure: normative values in an Italian population sample. Neurol Sci. 2002 Mar;22(6):443-7. doi: 10.1007/s100720200003. PMID: 11976975.

8. Spinnler H and Tognoni G. "Standardizzazione e taratura italiana di test neuropsicologici. Vol. Supplementum N. 8." (1987).
